# Therapeutic Plasma Exchange as Management of Complicated Systemic Lupus Erythematosus and Other Autoimmune Diseases

**DOI:** 10.1155/2019/5350960

**Published:** 2019-03-11

**Authors:** David Aguirre-Valencia, Juan Naranjo-Escobar, Iván Posso-Osorio, María Carmenza Macía-Mejía, Ivana Nieto-Aristizábal, Tatiana Barrera, María Alejandra Obando, Gabriel J. Tobón

**Affiliations:** ^1^Grupo de Investigación en Reumatología, Autoinmunidad y Medicina Traslacional (GIRAT), Fundación Valle del Lili and Universidad Icesi, Cali, Colombia; ^2^Blood Bank and Transfusion Service, Fundación Valle del Lili, Cali, Colombia; ^3^Laboratory of Immunology, Fundación Valle del Lili, Cali, Colombia

## Abstract

**Introduction:**

Autoimmune diseases include a diverse and complex group of pathologies with a broad clinical spectrum due to the production of autoantibodies, which generates multisystemic compromise. Therapeutic plasma exchange (TPE) is a good additive treatment for immunosuppression due to its action over the autoantibodies.

**Objectives:**

To describe the main clinical characteristics and outcomes of patients with systemic lupus erythematosus and other systemic autoimmune diseases managed with TPE.

**Methodology:**

This descriptive retrospective study enrolled patients with systemic autoimmune diseases who received TPE.

**Results:**

In total, 66 patients with a median age of 33.5 years (24-53 years) were included; the majority were females [n=51 (77.27%)]. Forty (60.61%) patients were diagnosed with systemic lupus erythematosus. In these cases, the main indication for TPE was diffuse alveolar hemorrhage (DAH; n=20, 30.3%) and neurolupus (n=9, 13.6%). No TPE-related deaths occurred, and the main complication was hemorrhage, without significant differences among the four types of TPE solutions used. The overall outcome was improvement in 41 (62.12%) patients.

**Conclusion:**

TPE is safe and effective in patients with severe manifestations of autoimmune diseases.

## 1. Introduction

Autoimmune diseases include a broad spectrum of pathologies, compromising diverse organs, tissues, systems, or, in some cases, systemic involvement, and these affect up to 7% of the population worldwide. The pathophysiology of these diseases can be mediated by both cellular and humoral immunity. When there is an exaggerated production of autoantibodies against certain antigens, damage is induced and immune complexes are formed, increasing organ injury, which is irreversible in some cases if timely interventions are not performed [1]. Among the group of autoimmune diseases with severe multisystemic compromise, systemic lupus erythematosus (SLE); antiphospholipid syndrome (APS); ANCA-positive vasculitis, such as granulomatosis with polyangiitis (previously known as Wegener's granulomatosis), microscopic polyangiitis (MPA), and eosinophilic granulomatosis with polyangiitis (previously known as Churg-Straus syndrome); autoimmune hemolytic anemia (AHAI); idiopathic thrombocytopenic purpura (ITP); and systemic sclerosis (scleroderma) are well described. Although all these entities are treated with different immunosuppressive agents, each with different levels of evidence and outcomes, when there is severe compromise, positive outcomes have been shown following the removal of autoantibodies with therapeutic plasma exchange (TPE) procedure in critical patients [2–4]. The American Society for Apheresis (ASFA) guidelines of 2016, define the term plasmapheresis as “a procedure in which blood of the patient or the donor is passed through a medical device which separates plasma from other components of blood and the plasma is removed (i.e., less than 15% of total plasma volume) without the use of colloid replacement solution” and TPE as “a therapeutic procedure in which blood of the patient is passed through a medical device which separates plasma from other components of blood. The plasma is removed and replaced with a replacement solution such as colloid solution (e.g., albumin and/or plasma) or a combination of crystalloid/colloid solution” [4].

TPE is an extracorporeal blood purification technique for the removal of high molecular weight substances (>15,000 Da), such as pathogenic autoantibodies, immune complexes, cryoglobulins, myeloma light chains, endotoxins, and lipoproteins that contain cholesterol. The basic premise of this treatment is to reverse the pathological process mediated by these substances. Other potential benefits of TPE include the discharge of the reticuloendothelial system, the stimulation of lymphocyte clones to increase cytotoxic therapy, and the possibility of reinfusion of large amounts of plasma without the risk of intravascular volume overload [2, 5, 6]. Although this technique has been used and has been proven to be a good therapeutic option, evidence is still scarce. The objective of this study was to describe the demographic and clinical characteristics, whether the election treatment was TPE, and the outcome of patients with systemic autoimmune diseases who received either of those management options at a high-complexity center.

## 2. Methods

This descriptive retrospective study enrolled every patient, male and female that met the inclusion criteria of receiving TPE as treatment for systemic autoimmune diseases (SLE, ANCA-associated vasculitis, inflammatory myopathies, diffuse scleroderma, autoimmune meningoencephalitis, rheumatoid arthritis, cryoglobulinemia, and primary APS), at the Fundación Valle del Lili, which is a high-complexity hospital in Cali-Colombia, from January 1, 2011, to May 31, 2017. Patients with solid organ and hematopoietic neoplasia, myasthenia gravis, acute demyelinating polyneuropathy (Güillain–Barré syndrome), and chronic idiopathic demyelinating polyneuropathy (CIDP), who received TPE, as well as those with incomplete clinical records were excluded. This study was approved by the ethics committee in Fundación Valle del Lili, which accepted the nonperformance of patient consent.

### 2.1. Procedures

TPE was performed by continuous flow centrifugation (Fenwal Amicus®; Com.Tec Fresenius Kabi®). The venous access was always central line. As separation is achieved by centrifugation, patients receive regional anticoagulation with citrate, which varies in proportion from 1:12 to 1:16 as well as the replacement solution, depending on the volume, time and type of solution for each subject. Prophylactic calcium was not part of the protocol. Plasma exchanges were performed with 5% albumin, fresh frozen plasma (FFP), 5% albumin combined solution and FFP, or 4% polysuccinate gelatin (Infukoll®), according to the indication of the attending physician and intensive care group. The procedures were performed by trained nurses in charge of apheresis and hemodialysis.

Descriptive statistical analysis was conducted, and continuous variables were expressed as average and standard deviation or median and interquartile range (IQR) according to the assumption of normality. Categorical variables were presented in proportions, and the Chi-square test or Fisher's exact test was performed to determine the correlation between them as appropriate. Statistical significance was defined as p < 0.05. Statistical software STATA 12.1 was used to analyze the data. This study was approved by the ethics committee.

## 3. Results

### 3.1. Demographic Characteristics

This study included 66 patients who met the inclusion criteria, of which 51 (77.3%) were women and 15 (22.7%) were men. The average age was 33.5 years (range, 24–53 years) and the majority of patients were Hispanic (n=63, 95.45%).

### 3.2. Diagnosis of Autoimmune Disease and TPE Indication

Table 1 shows the most frequent pathologies and main indications of TPE. The most prevalent diagnosis was SLE (n=40, 60.61%). In these patients, the main indication of TPE was diffuse alveolar hemorrhage (DAH; n=11, 27.5%) and neuropsychiatric involvement (n=9, 22.5%), including patients with central, peripheral, and psychiatric compromise. The other indications in patients with SLE were thrombotic microangiopathy (n=5, 12.5%), catastrophic APS (n=4, 10%), lupus nephritis (n=4, 10%; all had type IV diffuse proliferative nephritis), gastrointestinal involvement (lupus pseudo-obstruction; n=1, 2.5%), severe SLE (polyserositis and joint swelling; n=1, 2.5%), severe resistance to type B insulin (n=1, 2.5%), Evans syndrome (n=1, 2.5%), and kidney allograft rejection (n=1, 2.5%). Two additional patients (5%) received treatment with TPE due to severe cutaneous involvement, one of which had refractory pyoderma gangrenosum.

The second most prevalent diagnosis was ANCA-associated vasculitis, type microscopic polyangiitis (MPA) in eight cases (12.12%). The indications in these patients were DAH (n = 4, 50%) and rapidly progressive glomerulonephritis (RPG; n=4, 50%). The third most prevalent diagnosis was ANCA vasculitis type granulomatosis with polyangiitis (n=4, 6.06%). The indications in these patients were alveolar hemorrhage and RPG (n=2, 50%, each). There were two cases with diffuse scleroderma (3.03%), two with autoimmune meningoencephalitis (3.03%), and two with polymyositis (3.03%).  Table 2 shows the indication of TPE and the solution used for plasma exchange.

### 3.3. Medications Received

The majority of patients were receiving immunosuppressive treatment at the time of TPE, including chloroquine or hydroxychloroquine (n=11, 16.6%), prednisone or prednisolone (n=43, 65%), and methylprednisolone pulses during the same hospitalization in which TPE was indicated (n=33, 50%). Twenty-seven patients (41%) received or had received cyclophosphamide during hospitalization, with an average dose of 1.53 grams. Sixteen patients (24%) also received or had received Rituximab.

The replacement solutions for plasma exchange were 5% albumin (n=39, 59%), 5% albumin + FFP (n=8, 12.1%), 4% polysuccinate gelatin (n=10, 15.1%), and FFP (n=9, 13.6%). The average volume of TPE was 2237 ml for 5% albumin; 1988 ml for FFP; 3334 ml for 5% albumin combined solution and FFP; and 2150 ml for 4% polysuccinate gelatin (Infukoll®). Furthermore, the average number of sessions of TPE per patient was 5.39 sessions (range, 3-14). There were no significant differences in complications and outcomes among the four types of plasma replacement solutions.

The main indication of TPE was DAH in 30.3% of all cases. In these patients, the combination of 5% albumin + FFP was used in 35% of cases, 5% albumin in 40%, gelatin in 15%, and FFP in 10%.

### 3.4. Complications and Outcomes

Table 3 summarizes the main complications associated with TPE. No TPE-related deaths occurred. The majority of these were mild, with bleeding (n=17, 25.8%) being the most common. None of the cases presented hemodynamic instability. Additionally, hydroelectrolytic disorders were present in four (6.1%) patients, hypotension in six (9.1%), mild arrhythmias in two (3%), and hypersensitivity in one (1.5%). Eighteen patients (27.3%) developed infections in the 14 days after TPE; however, only two patients (3%) developed an infection associated with the Mahurkar catheter used for plasma exchange and/or hemodialysis.  Table 4 summarizes the most frequent indications and outcomes of our cases. Of the patients with DAH, 11 showed improvement, two (10%) had no improvement, and seven (35%) worsened despite TPE.

## 4. Discussion

Here, we report the largest case series of TPE in patients with systemic autoimmune diseases, being SLE the most representative diagnosis of the study sample in a single Latin American center. There are approximately 179 indications in which TPE has been used, with different outcomes and levels of evidence according to the ASFA guidelines [4]. It is most likely that immune-mediated diseases are not explained by a single high molecular weight substance, such as autoantibodies. However, despite limited evidence, the clinical improvement of patients in our series was 63.6%, which is similar to other published series [7, 8].

TPE is an invasive procedure with an acceptable risk/benefit ratio, when it is performed in accepted indications and managed by trained doctors and nurses [9]. According to the registry of the World Apheresis Association (WAA), in apheresis treatments in general, adverse events are classified as mild if tolerated without medication, moderate if they require medication, severe whereby treatment should be interrupted, and death [10]. In patients with autoimmune diseases, the highest rate of adverse events corresponds to moderate with 5.3%. The most common mild adverse events in the WAA registry were venous access problems and hypotension, from the moderate adverse events, paresthesia, and urticaria and from the severe ones, hypotension, and syncope. Regarding the replacement component, moderate adverse events were greater with FFP (6%). In some studies death has been reported in up to 0.05% cases [11], compared to our study, where no patient died secondary to TPE as a direct cause; however, 18.2% died due to progression of the underlying autoimmune disease. Most complications during TPE were mild; bleeding was greater in patients with albumin replacement than in those who received FFP; despite that, no significant differences were found among the four types of replacement solutions used. The majority of patients were admitted to the ICU with prolonged hospitalization and were administered immunosuppressive therapy, whereby 27.3% patients developed infections 14 days after TPE.

In terms of benefits according to each replacement solution, 5% albumin can decrease the risk of viral infections and anaphylactic reactions [3, 5, 6]; FFP contains all the noncellular components of normal blood and does not lead to postapheresis coagulopathy or immunoglobulin depletion [2, 4, 6]; and plasma expanders based on synthetic gelatins have been used safely in TPE with fewer changes in coagulation, but with similar changes in immunoglobulin levels as observed with albumin [3, 12, 13]. A study published in 2015 showed that patients who received albumin as replacement fluid and those in whom prophylactic sodium bicarbonate was not performed presented more adverse events [14].

DAH is a catastrophic clinical syndrome with an average mortality rate of 50% [15]. The main causes are ANCA vasculitis type granulomatosis with polyangiitis (32%), Goodpasture syndrome (13%), and MPA (9%) [16]. The frequency of DAH associated with SLE is variable, with rates ranging from 0.6% to 5.7%, and may be the initial manifestation in 10–20% of cases [16–18]. In a series by Kim et al. with 47 SLE and DAH patients, TPE was performed in 33% of the cases, resulting in a mortality rate of 29.2%; TPE was described as a factor associated with death with an odds ratio of 7.62. However, these were patients had greater disease severity [19]. Previously, we reported seven patients with SLE and DAH; four received plasma exchange and stabilization was performed in all cases but one, who died after DAH recurrence [16]. The use of plasma exchange in SLE and DAH case series varies between 5.9 and 66.6% [17], yet there are no systematic reviews on the type of solution most suitable for plasma exchange in such patients.

There are multiple neuropsychiatric manifestations of SLE (NPSLE), which are mostly severe with a poor prognosis. The frequency varies between 14 and 75%, with high mortality rates despite aggressive immunosuppressive therapy, which is why TPE has been described as a safe and effective alternative in severe cases [20]. Neuwelt et al. reviewed 26 patients with SLE and CNS involvement treated with TPE alone or in combination with cyclophosphamide. After therapy, 74% patients improved, 13% stabilized, and 13% progressed. Bartolucci et al. described 13 patients with NPSLE treated with TPE, whereby 54% showed complete remission and 46% partial remission [21–23]. In our series, of the nine patients with NPSLE, seven improved (77%), one patient showed no change, and one patient died from pneumonia.

Regarding APS, up to 50% of patients with SLE have antiphospholipid antibodies, one-third will develop clinical manifestations, and 1% are at risk of presenting catastrophic APS (CAPS), which has a mortality rate of 48% [24]. The highest rates of recovery (77.8%) have been achieved with the combination of anticoagulation, corticosteroids, and TPE. The increased use of these therapies since 2001 has resulted in a 20% reduction of mortality, with a mortality rate of 39% [25, 26]. In a 230 CAPS case series, treatment with TPE was independently associated with decreased mortality (OR = 0.36, 95% confidence interval, 0.14–0.92; p = 0.033) [27]. In the CAPS registry, plasma exchange was performed in 181 of 522 (35%) cases. Of 150 patients with SLE and CAPS, 48 died, and of 340 cases with primary CAPS, 33 died [28]. In our series, of five patients with CAPS, four improved and one died. At present, there is no consensus regarding the type of solution for replacement in CAPS [7].

Thrombotic microangiopathy associated with thrombocytopenic purpura (TTP) has been reported in 0.5–22% of SLE patients, with a higher frequency of 35–50% in juvenile SLE patients [29]. Furthermore, this complication occurs in 14% of patients with associated APS [28]. The standard treatment is TPE combined with corticosteroids, cyclophosphamide, IVIG, and anticoagulation. The use of TPE dramatically decreased mortality to 10–20%. However, in this series, of five patients with thrombotic microangiopathy, two improved, one did not change, and two died (40%).

The findings of studies on TPE use in lupus nephritis are variable, with a rapid decrease in proteinuria at short-term; however, the long-term results are similar to standard therapy with immunosuppressants [30–32]. In our series, of the four patients with type IV LN, 50% improved, and 50% worsened and remained in renal replacement therapy. In one of the two patients with severe cutaneous manifestations due to SLE, a refractory pyoderma gangrenosum was diagnosed; he received 13 sessions of TPE with little improvement. In the literature, 10 cases of pyoderma gangrenosum have received TPE, with very variable responses [33].

There are few reports of type B insulin resistance associated with SLE; however, there is evidence of the benefit of TPE in this rare autoimmune entity [34], such as a patient who showed improvement with the addition of Rituximab. Additionally, a patient with gastrointestinal involvement secondary to SLE due to lupus pseudo-obstruction was refractory to multiple managements, including plasma exchange.

Regarding ANCA-associated vasculitis, TPE is part of the standard treatment due to the positive evidence supporting its use. The MEPEX trial showed that TPE is associated with an increase in renal recovery, a reduction in risk of end-stage renal disease (ESRD), and reduced dependence on hemodialysis compared with methylprednisolone in patients with renal failure and ANCA vasculitis [34]. TPE is recommended to treat severe AAV, defined as severe acute renal failure (serum creatinine > 500 *μ*mol/L or hemodialysis), and/or DAH; seven sessions in a period of 14 days with a volume of 60 mL/kg/session are recommended [35, 36].

The fatal outcomes of TPE versus immunosuppression in renal involvement due to ANCA vasculitis are not significant different [37, 38]. In a recent Latin American study, Caffagi et al. compared 48 patients with ANCA vasculitis (GPA and MAP), whereby 24 patients received plasma exchange and the other 24 immunosuppressive therapy only. After 12 months, both groups showed eGFR improvement, and the survival rate was 79% in the plasma exchange group and 96% in the control group; the main cause of death was infections [39]. Regarding DAH secondary to ANCA vasculitis and Goodpasture syndrome, there are no randomized trials on the use of TPE. Klemmer et al. analyzed 20 cases of ANCA vasculitis with DAH and showed that alveolar hemorrhage was resolved in 20 patients with an average of 6.4 plasma exchange sessions, 55% of the 20 DAH patients improved, and 35% died [40].

In our study, two cases of diffuse scleroderma received plasma exchange due to DAH and severe fibrous and cutaneous involvement with rapid progression. The DAH associated with systemic sclerosis is very controversial; few cases have been reported [41]. Cozzi et al. performed TPE in 28 patients with SSc compared to 25 controls with D-penicillamine, the TPE regimen used was 4% albumin every 2–3 days in the first two weeks, then once a week for three months, and finally once every two weeks as a maintenance regimen. Serum aminoterminal type III procollagen peptide and interleukin 2 soluble receptor levels and DR+ T cells blood percentages before and after TPE were measured, in addition to skin and visceral scores. They concluded that there was a statistically significant decrease in the serum levels of these substances due to disease progression, as well as the clinical scores [42]. In our series, neither of the two patients improved their signs and symptoms related to scleroderma; however, alveolar hemorrhage was resolved in one patient.

None of the three patients with inflammatory myopathies improved. Consistent with similar evidence in the literature, the use of TPE in these entities would not be recommended, because in many cases they are mediated by cellular cytotoxicity and not by antibodies [7, 43].

Primary CNS vasculitis is a rare condition (2.4 cases per million people), with mortality rate of 10–17% and moderate to severe neurological sequelae in up to 20% of cases. Due to the lack of randomized clinical trials, treatment is based on other vasculitis and case reports, with pulses of steroids and cyclophosphamide [44]. In a series of 163 patients reported by Salvarani et al., two patients received TPE [45]. The only case in our series with this condition showed symptom improvement.

A case of type II cryoglobulinemia has been described, with excellent response to TPE and subsequent Rituximab with sufficient evidence in this entity [7, 46].

From our data, we can conclude that TPE is a safe procedure with good responses observed in patients with systemic autoimmune pathologies mediated by autoantibodies. The overall outcomes were improvement in 41 (62.12%) patients, with no changes in 13 (19.6%), and death in 12 (18%), none of which was secondary to TPE. More studies should be performed to determine the best solution to perform plasma exchange.

## Figures and Tables

**Table 1:** Autoimmune disease and therapeutic plasma exchange (TPE) indications.

*SLE*	*N=40 (60.61)*

DAH	11 (27.5)

Neuropsychiatric SLE	9 (22.5)

Thrombotic microangiopathy	5 (12.5)

CAPS	4 (10)

Lupus nephritis (WHO Class IV)	4 (10)

Severe skin lupus	2 (5)

Gastrointestinal lupus	1 (2.5)

Severe SLE	1 (2.5)

Type B insulin resistance	1 (2.5)

Evans syndrome	1 (2.5)

Rejection of the kidney allograft	1 (2.5)

*ANCA-associated vasculitis type Microscopic polyangiitis*	*8 (12.12)*

DAH	4 (50)

Rapidly progressive glomerulonephritis	4 (50)

DAH plus rapidly progressive glomerulonephritis	2 (25)

*ANCA-associated vasculitis type granulomatosis with polyangiitis*	*4 (6.06)*

DAH	2 (50)

Rapidly progressive glomerulonephritis	2 (50)

DAH plus rapidly progressive glomerulonephritis	1 (25)

*Inflammatory myopathies*	*3 (4.54)*

*Diffuse scleroderma*	*2 (3.04)*

DAH	1 (50)

Severe scleroderma	1 (50)

*Autoimmune meningoencephalitis*	*2 (3.03)*

*Rheumatoid arthritis*	*1 (1.52)*

*Cryoglobulinemia*	*1 (1.52)*

*Primary APS*	*1 (1.52)*

CAPS	1 (100)

*Goodpasture Syndrome*	*1 (1.52)*

DAH	1 (100)

*Devic syndrome*	*1 (1.52)*

*Vasculitis*	*1 (1.52)*

*CNS Primary Vasculitis*	*1 (1.52)*

*Average of therapeutic plasma exchanges∗∗*	5.39 (3-14)

*∗∗*Median and interquartile range (IQR).

SLE: systemic lupus erythematosus; DAH:diffuse alveolar hemorrhage; CAPS: catastrophic antiphospholipid syndrome; WHO: World Health Organization; APS: antiphospholipid syndrome; CNS: central nervous system.

**Table 2:** TPE indications and TPE replacement solutions.

TPE indications	TPE replacement solution, n (%)				Total, n=66
	Albumin 5%, n=39	Albumin 5% plus Fresh frozen plasma, n=8	4% succinylated gelatin, n=10	Fresh frozen plasma, n=9	
DAH	8 (20.51)	7 (87.5)	3 (30)	2 (22.22)	20 (30.3)
Neuropsychiatric SLE	5 (12.81)	1 (12.5)	2 (20)	1 (11.11)	9 (13.6)
Rapidly progressive glomerulonephritis	6 (15.38)	0 (0)	1 (10)	0 (0)	7 (10.6)
Thrombotic microangiopathy	1 (2.56)	0 (0)	1 (10)	3 (33.33)	5 (7.6)
CAPS	3 (7.69)	0 (0)	0 (0)	2 (22.22)	5 (7.6)
Lupus nephritis (WHO Class IV)	2 (5.13)	0 (0)	1 (10)	1 (11.11)	4 (6.1)
Severe skin lupus	2 (5.13)	0 (0)	1 (10)	0 (0)	3 (4.5)
Inflammatory myopathies	3 (7.69)	0 (0)	0 (0)	0 (0)	3 (4.5)
Gastrointestinal lupus	1 (2.56)	0 (0)	0 (0)	0 (0)	1 (1.5)
Severe scleroderma	1 (2.56)	0 (0)	0 (0)	0 (0)	1 (1.5)
Severe SLE	1 (2.56)	0 (0)	0 (0)	0 (0)	1 (1.5)
Autoimmune meningoencephalitis	1 (2.56)	0 (0)	0 (0)	0 (0)	1 (1.5)
Neuromyelitis Optical	1 (2.56)	0 (0)	0 (0)	0 (0)	1 (1.5)
Rejection of the kidney allograft	1 (2.56)	0 (0)	0 (0)	0 (0)	1 (1.5)
Type B insulin resistance	1 (2.56)	0 (0)	0 (0)	0 (0)	1 (1.5)
Evans syndrome	1 (2.56)	0 (0)	0 (0)	0 (0)	1 (1.5)
Legs vasculitis	0 (0)	0 (0)	1 (10)	0 (0)	1 (1.5)
CNS Vasculitis	1 (2.56)	0 (0)	0 (0)	0 (0)	1 (1.5)

TPE: therapeutic plasma exchange; DAH: diffuse alveolar hemorrhage; SLE: systemic lupus erythematosus; CAPS: catastrophic antiphospholipid syndrome; WHO: World Health Organization; CNS: central nervous system.

**Table 3:** Complications of therapeutic plasma exchange.

TPE complications/TPE replacement solutions	Albumin 5%, n=39	Albumin 5% plus Fresh frozen plasma, n=8	4% succinylated gelatin, n=10	Fresh frozen plasma, n=9	Total, n=66	p value
*Bleeding*	11 (28.2)	4 (50)	0 (0)	2 (22.2)	17 (25.8)	0.087
*Thrombosis*	0 (0)	0 (0)	0 (0)	0 (0)	0 (0)	0
*Hydroelectrolytic disorders*	1 (2.6)	2 (25)	1 (10)	0 (0)	4 (6.1)	0.067
*Hypotension*	1 (2.6)	1 (12.5)	2 (20)	2 (22.2)	6 (9.1)	0.055
*Allergies *	0 (0)	1 (12.5)	0 (0)	0 (0)	1 (1.5)	0.121
*Arrhythmias*	1 (2.6)	0 (0)	1 (10)	0 (0)	2 (3)	0.655
*Infections 14 days posterior to TPE*	5 (12.8)	6 (75)	3 (30)	4 (44.4)	18 (27.3)	0.002
*Catheter-Related Infections*	0 (0)	0 (0)	1 (10)	1 (11)	2 (3)	
*Type of infection*						
Bacterial infection	4 (80)	5 (83.3)	3 (100)	4 (100)	16 (88.9)	
Bacterial and Mycobacterial	0 (0)	1 (16.7)	0 (0)	0 (0)	1 (5.6)	
infection						
Viral infection	1 (20)	0 (0)	0 (0)	0 (0)	1 (5.6)	

TPE: therapeutic plasma exchange.

**Table 4:** TPE indications and outcomes.

TPE indication/Outcome	Improvement, N. patients (%)	Unchanged, N. patients (%)	Worsening, N. patients (%)	Total	Death, N. patients (%)	Dialysis
DAH	11 (55)	2 (10)	7 (35)	20	7 (35)	3 (15)
Neuropsychiatric SLE	7 (78)	1 (11)	1 (11)	9	1 (11)	1 (11)
RPGN	5 (71)	2 (29)	0	7	0	3 (60)
CAPS	4 (80)	0	1 (20)	5	1 (20)	0
Thrombotic microangiopathy	2 (40)	1 (20)	2 (40)	5	2 (40)	1 (20)
Lupus Nephritis	2 (50)	2 (50)	0	4	0	2 (50)
Lupus severe skin involvement	3 (100)	0	0	3	0	0
Inflammatory myopathies	1 (33)	1 (33)	1 (33)	3	1 (33)	0
Optic Neuromyelitis	1 (100)	0	0	1	0	0
Severe scleroderma	0	1 (100)	0	1	0	0
Evans Syndrome	0	1 (100)	0	1	0	0
Insulin resistance type B	1 (100)	0	0	1	0	0
Legs vasculitis	1 (100)	0	0	1	0	0
CNS Vasculitis	1 (100)	0	0	1	0	0
Gastrointestinal lupus	0	1 (100)	0	1	0	0
Autoimmune meningoencephalitis	1 (100)	0	0	1	0	0
Severe SLE	1 (100)	0	0	1	0	0
Acute allograft rejection	0	1 (100)	0	1	0	1 (100)

TPE: therapeutic plasma exchange; DAH: diffuse alveolar hemorrhage; SLE: systemic lupus erythematosus; RPGN: rapidly progressive glomerulonephritis CAPS: catastrophic antiphospholipid syndrome; CNS: central nervous system.

## Data Availability

The data used to support the findings of this study are available from the corresponding author upon request.

## Additional Points

*Study Limitations. *Although our study sample is relevant, with the enrollment of patients with any of the systemic autoimmune diseases, the sample for each one diminishes their representative attribute, whereby obtaining definitive conclusions for every pathology is limited. Other limitations are that it was a retrospective study, the lack of some data, and having a better antibody profile before and after TPE.
